# FDA Approves First Targeted Treatment for Cerebrotendinous Xanthomatosis: A Perspective on a Landmark in Rare Lipid Storage Disease Therapy

**DOI:** 10.1002/hsr2.71549

**Published:** 2025-11-26

**Authors:** Laiba Jalal, Areeba Aamir Ali Basaria, Hermann Yokolo

**Affiliations:** ^1^ Dow University of Health Sciences Karachi Pakistan; ^2^ Department of Research Medical Research Circle (MedReC) Goma Democratic Republic of the Congo

**Keywords:** cerebrotendinous xanthomatosis, chenodeoxycholic acid, cholestanol, CYP27A1 mutation, orphan disease

## Abstract

**Background and Aims:**

Cerebrotendinous xanthomatosis (CTX) is a rare autosomal recessive disorder caused by mutations in the CYP27A1 gene, leading to deficient sterol 27‐hydroxylase activity. This enzyme is critical for bile acid synthesis, and its dysfunction results in reduced chenodeoxycholic acid (CDCA) levels and subsequent accumulation of cholestanol in tissues. This study highlights the recent FDA approval of Ctexli (chenodiol), a synthetic CDCA formulation, as the first treatment option for CTX.

**Methods:**

A comprehensive literature search was performed using PubMed and Google Scholar, and relevant articles were identified that focused on the mechanism of action, clinical efficacy, and safety of chenodiol.

**Results:**

Data from the RESTORE Phase 3 trial demonstrated that chenodiol significantly reduced plasma cholestanol and urinary bile alcohol levels, with the most pronounced results observed in patients treated before the onset of irreversible neurological damage. The FDA approval of Ctexli validated these findings and established chenodiol as a reliable therapeutic option for CTX.

**Conclusion:**

The FDA approval of chenodiol marks a significant milestone in the management of CTX, providing a standardized and evidence‐based therapy for a previously neglected condition. Early diagnosis, broader screening, and global access to the drug will be key to improving outcomes and ensuring lasting clinical benefit.

## 1 Introduction

Cerebrotendinous xanthomatosis (CTX), also known as cerebral cholesterinosis, is a rare, autosomal recessive, lipid storage disorder caused by mutations in the *CYP27A1* gene. This gene codes for the enzyme sterol 27‐hydroxylase, which is essential for cholesterol metabolism and bile acid synthesis. Reduction in the activity of this enzyme leads to decreased formation of chenodeoxycholic acid (CDCA). CDCA exerts a negative feedback and decreases the formation of cholestanol, a cholesterol metabolite. However, due to reduced CDCA, formation of cholestanol is increased and it deposits in multiple tissues, including eye lenses, tendons, and brain, leading to disease manifestations such as cataract, xanthomas, and progressive neurological dysfunction, including peripheral neuropathy, cognitive impairment, and cerebellar ataxia [1, 2]. The presence of any two of these four symptoms: premature cataracts, diarrhea, progressive neurologic signs, and tendon xanthomas should prompt thorough biochemical evaluation for CTX. Elevated serum cholestanol levels function as a diagnostic marker along with typical radiological findings, which are symmetrical abnormalities in the dentate nucleus, as shown by T2W and FLAIR brain MRI [1].

## 2 Therapeutic Management and Recent Approval of Chenodiol

The most effective treatment option for CTX currently is chenodiol, which is a formulation of CDCA. When chenodiol levels are increased in the enterohepatic bile acid pool, the *CYP7A1* gene is downregulated, which leads to a reduction in the levels of cholestanol and urine 23S‐Pentol (a bile alcohol which accumulates abnormally in patients with CTX). Although CDCA (chenodiol) has proven to be effective in CTX patients, its use was historically limited to off‐label access before formal regulatory approval. Before regulatory approval, CDCA was available only through off‐label or compassionate use in many countries. This led to heterogeneous dosing strategies and poorly tracked long‐term outcomes. However, in February 2025, the FDA approved Ctexli (chenodiol) tablets for the treatment of CTX. This approval was based on data from the RESTORE trial, which was a randomized, crossover, double‐blind, placebo‐controlled trial evaluating the safety and efficacy of CDCA in patients with CTX [3]. The trial enrolled 14 genetically confirmed CTX patients (aged 18–75), stratified by neurological status, out of which 13 were randomized and 12 completed the treatment. The trial comprised 4 treatment phases, which included two open‐label phases, each lasting for 8 weeks, followed by two placebo‐controlled phases, each lasting for 4 weeks. Over 24 weeks, participants received 250 mg of CDCA orally three times daily. The primary endpoint was change in the urine 23S‐pentol levels, while secondary endpoints included normalization of 7α‐hydroxy‐4‐cholesten‐3‐one (7αC4) and changes in plasma cholestanol levels. At the end of the placebo‐controlled period, a 20‐fold increase (CI: 10.3, 43.5; *p* < 0.0001) was observed in the urine 23S‐pentol levels. Similarly, plasma cholestanol levels demonstrated a 2.8‐fold increase (CI: 1.5, 5.2; *p* = 0.0083) and plasma 7αC4 levels demonstrated a 50‐fold increase (CI: 25.0, 66.7; *p* < 0.0001) with chenodiol compared to placebo, highlighting CDCA's role in controlling this key disease biomarker [4]. The most commonly reported adverse events were diarrhea, headache, constipation, hypertension, muscle weakness, and upper respiratory tract infection. The prescribed regimen for CTEXLI is 250 mg taken orally three times per day [3].

## 3 Future Directions and Global Implications

The availability of an FDA‐approved therapy creates powerful motivation for clinicians to consider CTX in their differential diagnoses and pursue early detection. Research by Yahalom et al. has demonstrated that early intervention significantly alters disease trajectory, with patients receiving treatment before advanced neurological symptoms showing markedly better outcomes than those with delayed diagnosis [5]. The challenges of CTX diagnosis are magnified in resource‐limited settings such as Pakistan, where awareness of rare genetic disorders remains limited and access to specialized diagnostic testing is restricted. Contributing factors include limited genetics expertise, financial barriers to specialized testing, and the nonspecific nature of early CTX symptoms. The diagnosis is frequently made only after irreversible neurological damage has occurred, when characteristic but late‐appearing manifestations such as tendon xanthomas become evident. This pattern of delayed diagnosis significantly impacts treatment efficacy, as neurological damage often proves irreversible even with appropriate therapy [6].

With an approved treatment demonstrating clinical benefit, CTX now meets a crucial criterion for consideration in expanded newborn screening panels: the availability of effective intervention that substantially changes outcomes when initiated early. The scientific groundwork for such screening already exists, with methodologies developed for detecting elevated cholestanol in dried blood spots (DBS) and genetic testing for common *CYP27A1* mutations [7]. Integrating these techniques into existing screening infrastructure appears technically feasible and potentially cost‐effective given the significant morbidity prevention through early intervention. A study conducted by Hong et al. also concluded that DBS screening in newborns based on the concentration of metabolites (5β‐cholestane‐3α,7α,12α,25‐tetrol glucuronide (GlcA‐tetrol), the ratio of GlcA‐tetrol to tauro‐CDCA (t‐CDCA) (GlcA‐tetrol/t‐CDCA), and the ratio of tauro‐trihydroxycholestanoic acid (t‐THCA) to GlcA‐tetrol (t‐THCA/GlcA‐tetrol)) is useful and has an exceptionally low false‐positive rate [8].

The economic argument for screening becomes considerably stronger with an FDA‐approved therapy that provides standardized treatment protocols and outcomes measurement. Healthcare systems can now more confidently project the benefits of early identification against screening and treatment costs, potentially justifying the inclusion of CTX in routine newborn screening panels in developed countries with established screening programs. Despite few comprehensive cost‐effectiveness analyses, early detection by modalities such as DBS can significantly alleviate permanent neurological morbidity and the costs associated with it. Phased, regionally centralized implementation is suggested as a feasible approach to aid cost‐effectiveness and long‐term sustainability in settings of low‐ and middle‐income countries by embedding databases within health systems [9]. The FDA approval and orphan drug designation for chenodiol create potential pathways for inclusion in international humanitarian drug access programs. Such programs have successfully increased the availability of treatments for other rare disorders in regions where commercial markets alone would not support distribution [10].

Despite its promise, certain limitations persist. CDCA therapy yields maximal benefit when initiated early; in patients with established neurological deficits, reversal is often incomplete [5]. Long‐term safety beyond 2 years remains insufficiently studied in diverse populations. Moreover, access to CDCA may remain limited in under‐resourced settings due to cost and regulatory barriers unless addressed through global access programs and public–private partnerships.

## 4 Conclusion

The approval of Ctexli represents a watershed moment for patients with CTX, a historically neglected disorder with devastating consequences when left untreated. Unlike previous therapeutic approaches, this FDA‐approved treatment provides a standardized, regulated, and evidence‐based intervention with established efficacy parameters. The approval's significance extends beyond treatment availability to its potential impacts on diagnosis rates, screening initiatives, and global rare disease management. While the recent development represents a major milestone, further clinical studies are necessary to evaluate the long‐term safety and efficacy of chenodiol across diverse patient populations.

## Author Contributions

**Laiba Jalal:** conceptualization; project administration; methodology; supervision; writing – original draft; writing – review and editing; literature search. **Areeba Aamir Ali Basaria:** conceptualization; project administration; methodology; writing – original draft; writing – review and editing; Literature search. **Hermann Yokolo:** methodology; writing – original draft; writing – review and editing; resources; literature search. Final approval of manuscript: All authors have reviewed and approved the final version of this manuscript. Each author agrees to be accountable for all aspects of the work and ensures that any questions related to the accuracy or integrity of any part of the manuscript are appropriately investigated and resolved.

## Ethics Statement

The authors have nothing to report.

## Consent

The authors have nothing to report.

## Conflicts of Interest

The authors declare no conflicts of interest.

## Peer Review

Not commissioned, externally peer reviewed.

## Transparency Statement

“All authors have read and approved the final version of the manuscript, Hermann YOKOLO had full access to all of the data in this study and takes complete responsibility for the integrity of the data and the accuracy of the data analysis.”

“The lead author, that is, Laiba Jalal affirms that this manuscript is an honest, accurate, and transparent account of the study being reported; that no important aspects of the study have been omitted; and that any discrepancies from the study as planned (and, if relevant, registered) have been explained.”

## Data Availability

The authors have nothing to report.
